# Assessment of neonatal respiratory rate variability

**DOI:** 10.1007/s10877-022-00840-2

**Published:** 2022-03-25

**Authors:** Jesse Coleman, Amy Sarah Ginsburg, William M. Macharia, Roseline Ochieng, Dorothy Chomba, Guohai Zhou, Dustin Dunsmuir, Walter Karlen, J. Mark Ansermino

**Affiliations:** 1 Evaluation of Technologies for Neonates in Africa (ETNA), Nairobi, Kenya; 2 Clinical Trials Unit, University of Washington, Seattle, WA USA; 3 Department of Pediatrics, Aga Khan University, Nairobi, Kenya; 4 Center for Clinical Investigation, Brigham and Women’s Hospital, Boston, MA USA; 5 Anesthesiology, Pharmacology & Therapeutics, The University of British Columbia, Vancouver, BC Canada; 6 Mobile Health Systems Lab, Department of Health Sciences and Technology, ETH Zurich, Zurich, Switzerland; 7 Centre for International Child Health, 305 – 4088 Cambie Street, Vancouver, BC V5Z 2X8 Canada

**Keywords:** Child health, Critical care, Delivery of health care, Diagnosis, Patient care

## Abstract

Accurate measurement of respiratory rate (RR) in neonates is challenging due to high neonatal RR variability (RRV). There is growing evidence that RRV measurement could inform and guide neonatal care. We sought to quantify neonatal RRV during a clinical study in which we compared multiparameter continuous physiological monitoring (MCPM) devices. Measurements of capnography-recorded exhaled carbon dioxide across 60-s epochs were collected from neonates admitted to the neonatal unit at Aga Khan University-Nairobi hospital. Breaths were manually counted from capnograms and using an automated signal detection algorithm which also calculated mean and median RR for each epoch. Outcome measures were between- and within-neonate RRV, between- and within-epoch RRV, and 95% limits of agreement, bias, and root-mean-square deviation. Twenty-seven neonates were included, with 130 epochs analysed. Mean manual breath count (MBC) was 48 breaths per minute. Median RRV ranged from 11.5% (interquartile range (IQR) 6.8–18.9%) to 28.1% (IQR 23.5–36.7%). Bias and limits of agreement for MBC vs algorithm-derived breath count, MBC vs algorithm-derived median breath rate, MBC vs algorithm-derived mean breath rate were − 0.5 (− 2.7, 1.66), − 3.16 (− 12.12, 5.8), and − 3.99 (− 11.3, 3.32), respectively. The marked RRV highlights the challenge of performing accurate RR measurements in neonates. More research is required to optimize the use of RRV to improve care. When evaluating MCPM devices, accuracy thresholds should be less stringent in newborns due to increased RRV. Lastly, median RR, which discounts the impact of extreme outliers, may be more reflective of the underlying physiological control of breathing.

**Supplementary Information:**

The online version contains supplementary material available at 10.1007/s10877-022-00840-2.

## Introduction

Accurate measurement of respiratory rate (RR) in children is particularly important in low-resource settings where illness and deterioration are typically diagnosed based on a child’s clinical signs and symptoms [1–3]. To measure RR, the World Health Organization (WHO) recommends observing and counting chest and abdominal movements over a full 60 s [4]. In practice, this recommendation is frequently modified to counting respirations over a shorter duration of time (e.g., counting breaths for 10 s and multiplying by six). This modification can result in up to a 50% discrepancy compared to WHO recommendations [5]. In addition to inaccuracy, manual breath counting can be influenced significantly by counter bias and lacks reproducibility.

In neonates, measuring RR accurately is especially challenging given high RR and the within-neonate RR variability (RRV) [6]. Control of breathing remains immature until after the first month of life. Neonates often demonstrate periodic breathing, a benign, regular manifestation of irregular respiratory patterns, consisting of contiguous periods of alternating breaths and respiratory pauses. Healthy neonates also may exhibit benign irregularly irregular breathing patterns with short periods of apnea, similar to the disrupted respiratory control and ataxic breathing which is seen with opioid administration [7, 8]. These irregular breathing patterns are distinct from the regular rhythms of periodic breathing or the apnea of prematurity, though there appears to be some amount of overlap [9, 10]. In contrast to older infants and children, neonates have a marked degree of breath-to-breath variability and are also more likely to exhibit episodes of respiratory pause associated with stimulation and in response to hypoxia [11]. This individual-level, within-neonate RRV, adds to the complexity of identifying and reliably measuring true RR [12]. Perturbations, including sighs, swallows, and coughs, can affect the duration of individual breaths, the resulting RR, and the within-neonate RRV. Given the high RRV in neonates, quantifying RR by observing chest and abdominal movements as recommended by the WHO is fraught with potential inaccuracy [13].

There will always be some level of uncertainty when performing manual or automated measurement of RR. All potential sources for uncertainty in RR measurement should be considered (Table 1). The size and acceptability of the resulting uncertainty depend on the conditions and context of the measurement. In some clinical situations, high accuracy may not be necessary. However, in the emergency department or intensive care unit, when it is crucial to determine if a patient has crossed a diagnostic threshold, accurate RR measurement can enable early identification and expedited management of patient deterioration [14–17]. In research laboratories, accuracy and precision are essential for determining how a new device or method compares with the reference method.

**Table 1:** Potential sources of uncertainty in respiratory rate measurement, approximate distribution, and potential solutions

Uncertainty	Source	Distribution		Possible solution
Breath-to-breath variation	Neonate	Normal	Random	Extended within-neonate observation
Perturbation (e.g., sigh, swallow, cough)	Neonate	Multimodal	Non-random	Exclude outliers
Device uncertainty due to inaccurate timing	Device	Normal	Random	Repeatability with multiple simultaneous devices
Device uncertainty due to missed breath(s)	Device	Multimodal	Non-random	Repeatability with multiple simultaneous devices
Observer error due to inaccurate timing	Observer	Normal	Random	Repeatability with multiple simultaneous observers
Observer error due to missed breath(s)	Observer	Multimodal	Non-random	Repeatability with multiple simultaneous observers
Rounding error due to counting breaths in a time interval	Analysis	Normal	Random	Measure breath interval (not count)

The nature of the distributions is based on knowledge of the underlying processes and has not been validated

There is growing evidence which suggests including RRV in clinical scoring systems may be beneficial for guiding escalation and de-escalation of care [18, 19]. Heart rate variability (HRV), unlike RRV, has been extensively studied and is commonly used as a marker of risk for mortality [20–23]. HRV is considered to be central in the clinical assessment of diverse conditions that include neurological and sleep disorders, muscular dystrophy, and diabetes in adults, and sepsis in neonates [24, 25]. Like HRV, the identification of changes in RRV could be used as an indicator of underlying physiological disturbances [18, 19, 26].

Numerous innovative RR monitoring methods and devices for both adults and neonates using non-contact video, sensors embedded in bedding, motion sensors, nanoparticles, and temperature-based methods have been reported previously [27–31]. The variety of monitoring methods has resulted in various different evaluation methods and difficulty when trying to compare results across studies. A recent systematic review of RR monitoring systems suggested standardizing validation frameworks to directly compare different RR monitoring methods and systems [32]. Fortunately, detailed verification and validation recommendations have been made [33, 34]. If followed, these recommendations may result in future cross-comparable research of neonatal RRV. The current research is a result of data analyzed during systematic verification conducted within a device comparison study.

In our study comparing neonatal multiparameter continuous physiological monitoring (MCPM) devices in Nairobi, Kenya we sought to quantify RRV between- and within-neonates, as well as between- and within-epochs to identify the best methods for device comparison studies. We believe this RRV quantification will inform management of uncertainty and RRV when designing, developing, and comparing RR monitoring devices in neonates.

## Methods

### Setting and participants

We conducted a clinical verification phase of the reference RR monitoring device while studying low-cost neonatal MCPM devices in Nairobi, Kenya [35]. Study participants were spontaneously breathing neonates admitted for observation and care in the maternity ward, neonatal intensive care, and the neonatal high dependency units at Aga Khan University-Nairobi (AKU-N) Hospital. Caregivers were approached, recruited, and sequentially screened for enrollment by trained study staff during routine intake procedures. Final eligibility determination was based on medical history, physical examination, appropriate understanding of the study by the caregiver, and completion of the written informed consent process (Table 2).

**Table 2:** Study definitions and eligibility criteria

*Study definitions*		
Epoch	A 60-s period of time	
Breath	One cycle of neonate-initiated inhalation and exhalation (Table 3)	
Breath duration	Length of time from the start to the end of a single breath	
Breath start	End of a waveform trough (low point) where the carbon dioxide level starts to ascend	
Respiratory rate	Number of breaths initiated within an epoch	
Breath rate	The number of breaths within an epoch based on the median or mean of the breath duration	
Respiratory rate variability (RRV)	The dispersion of respiratory rate or breath duration (a reciprocal of the respiratory rate) within an epoch or between epochs, calculated as the standard deviation expressed as a percentage of the mean; epochs for comparison are defined below	
	Within-subject RRV	RRV_bd_ = Breath duration variability (average within each epoch) RRV_bm_ = Minute-to-minute variability (average between epochs) RRV_10bd_/RRV_60bd_ = Breath duration variability between epochs measured at 10- or 60-min intervals
	Between-subject RRV	RRV_bs_ = Between subject minute-to-minute variability
*Neonatal eligibility criteria*		
Inclusion criteria	∙ Male or female neonate, corrected age of < 28 days Willingness and ability of neonate’s caregiver to provide informed consent and to be available for follow-up for the planned duration of the study	
Exclusion criteria	∙ Receiving mechanical ventilation or continuous positive airway pressure ∙ Skin abnormalities in the nasopharynx and/or oropharynx ∙ Contraindication to the application of skin sensors ∙ Known arrhythmia ∙ Any medical or psychosocial condition or circumstance that, in the opinion of the investigators, would interfere with the conduct of the study or for which study participation might jeopardize the neonate’s health	

### Study procedures and data collection

Detailed study procedures are described in the published protocol [35]. In brief, term and preterm neonates were enrolled in a MCPM accuracy and feasibility evaluation. Male or female neonates were eligible if they had a corrected age of < 28 days and the caregiver was willing and able to provide informed consent and to be available for follow-up for the planned duration of the study. Neonates were excluded if they were receiving mechanical ventilation or continuous positive airway pressure, had skin abnormalities in the nasopharynx and/or oropharynx or a contraindication to the application of skin sensors, a known arrhythmia or any medical or psychosocial condition or circumstance that, in the opinion of the investigators, would interfere with the conduct of the study or for which study participation might jeopardize the neonate’s health. Solely for the purposes of the study, we used the Masimo Rad-97 with NormoLine capnography as a reference device to record and measure RR using exhaled carbon dioxide (CO_2_) levels. The collected continuous capnography data were digitized at approximately 20 Hz using asynchronous communication with a custom software application. Capnography readings were collected for a minimum of one hour and continued until the neonate was discharged (range 1–6.25 h; median: 3.75 h). Demographic and capnography data were entered and stored on a secure AKU-N-hosted REDCap server [36].

**Fig. 1:**
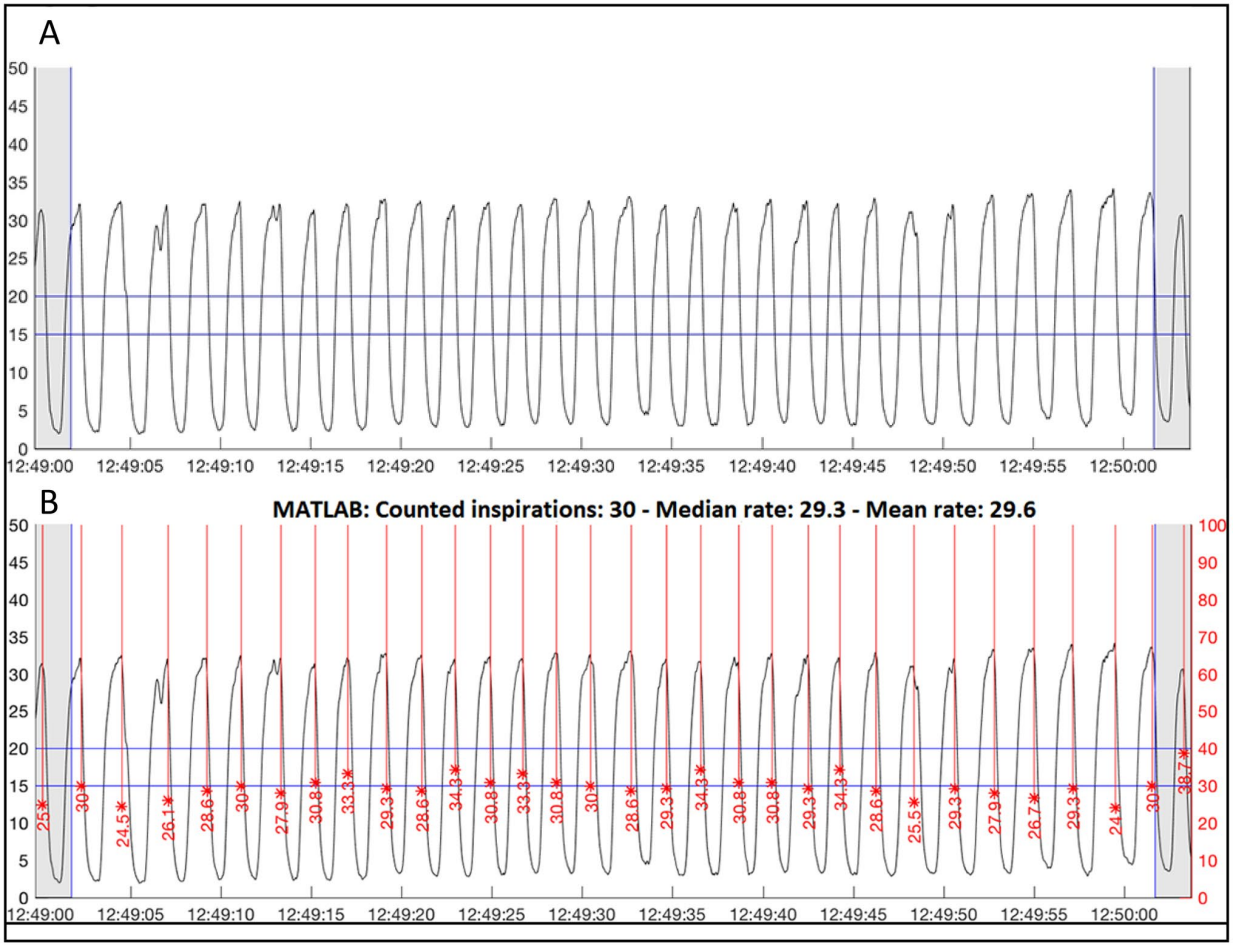
Example capnograms (carbon dioxide (CO_2_) waveform plots) before (**A**) and after (**B**) algorithm processing. The plotted CO_2_ waveform shows the breathing pattern of a neonate and algorithm-derived identification of breaths (red, vertical lines). Only peaks on the white background were included; peaks that fell within the grey zone were ignored as they were outside the 60-s epoch. **A** Plotted waveform from example epoch before processing by the algorithm. Each peak within the 60-s epoch was counted by one to three trained observers. The horizontal blue 15 and 20 lines were used to assist observers during irregular or incomplete breaths (not shown). **B** Plotted waveform after processing by algorithm. The red vertical lines show identified peaks, with the length and label of the red line representing the calculated breath rate based on the breath duration

Sixty-second epochs of capnography data were extracted at predetermined time intervals and converted to capnogram waveform tracings. Intervals between epochs were predetermined and based on study-related clinical observations: at 10-min intervals throughout the first hour of participation followed by 60-min intervals starting at the second hour [35]. The resulting capnogram tracings included a total of 64 s (Fig. 1); two seconds were added at the beginning and end of each epoch to facilitate manual breath counting of the epoch.

**Fig. 2:**
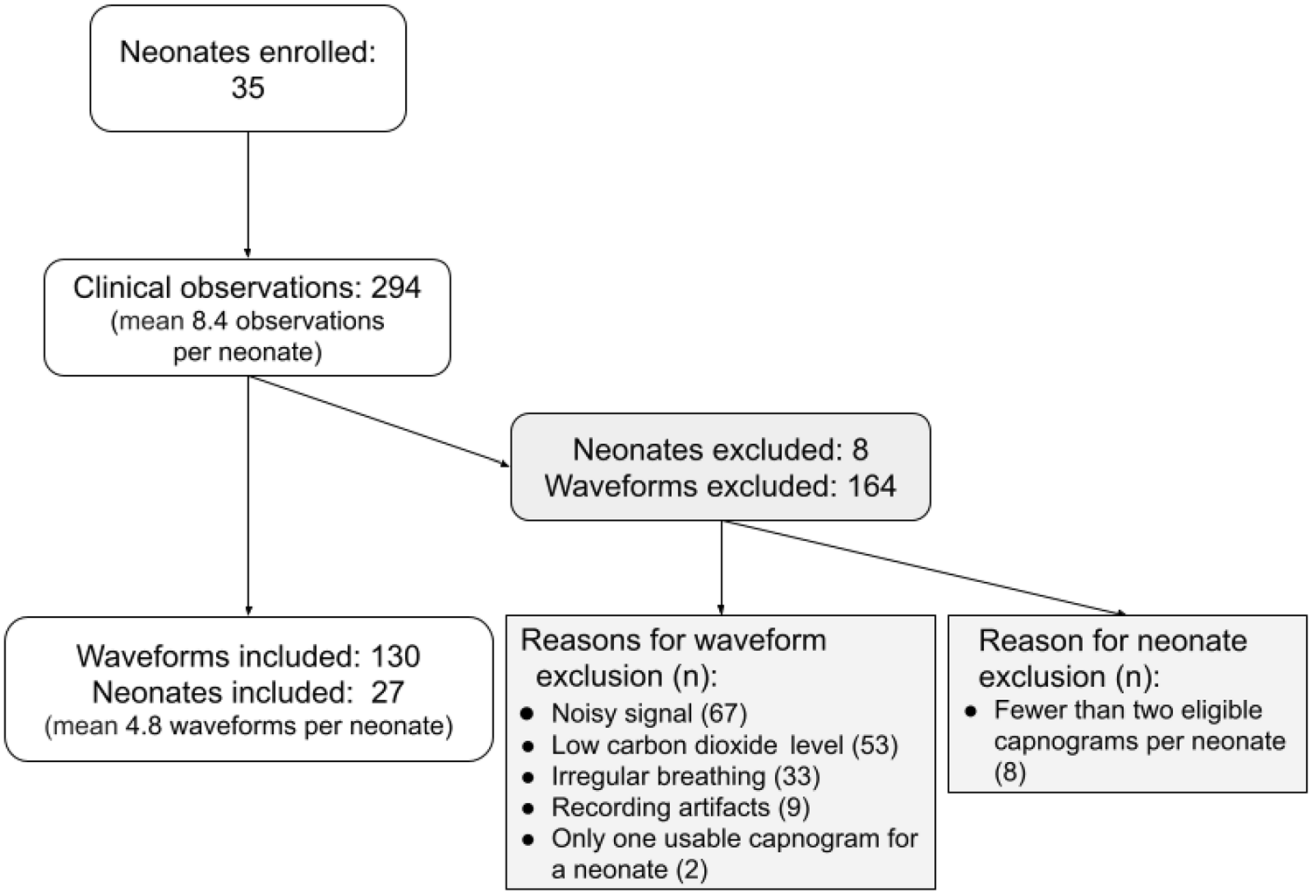
Recruitment flow diagram

One of the authors (JMA, an anesthesiologist) reviewed all capnogram tracings for quality control; difficult-to-count plots were discarded (n = 164; Fig. 2). All remaining epochs were included, and breaths were manually counted from capnograms and identified using an automated signal detection algorithm. For the manual counting, capnographs were provided to two trained observers to count all breaths within each epoch independently, and the results were averaged. A breath was identified using a set of predefined rules created by the investigators (Table 3). If the number of breaths counted by the two observers varied by more than three breaths per epoch, a third trained observer independently counted the plot, and the two closest counts were averaged.

**Table 3:** Rules for identifying breaths based on graphical waveform plots

1. Count peaks (tops) of the waveform that are within the white background. Ignore peaks that are within the grey background on either side of the image
2. A peak should be counted as a breath when the peak of the waveform is above 15 mmHg, the lower horizontal blue line
3. If the peak does not reach the lower horizontal blue line at 15 mmHg, to be counted as a breath, the peak should reach at least 50% of the mean peak
4. The waveform should dip down to the normal baseline (either below 15 mmHg, the lower horizontal blue line, or based on other breaths). If the waveform does not reach below this point, then this is considered part of the same (double) peak and only counted as a breath once

The automatic breath detection method was based on a time-domain algorithm that identified regular patterns in physiological waveforms [37].

The algorithm was modified to identify unique breaths in the CO_2_ signal by dividing the waveform in time and identifying changes in direction to define segments. Specifically, an incremental-merge algorithm divided the waveform into geometrically similar segments by establishing a line between equidistant samples and iteratively merging adjacent lines that had the same slope sign into single, longer lines. The resulting line segments of alternating slope signs defined inhalation and exhalation components, as well as artifacts interrupting this sequence. An adaptive threshold was applied to the length of these segments to separate artifacts and double breaths from regular breathing components [37].

The breath duration was calculated between the beginning of two adjacent regular inhalation components that were interrupted by at least one exhalation component and no artifact. To investigate the effect of length of time between epochs on breath duration variability (RRV_bd_), epochs were grouped into 10- and 60-min intervals for subgroup analysis.

### Data analysis

Results from the manual and automatic breath detection methods were analyzed using STATA 13 and R [38, 39]. The coefficient of variation, the standard deviation (SD) expressed as a percentage of the mean, was reported as RRV between- and within-neonates, as well as between- and within-epochs (see Table 2 for definitions).

Agreement between the manual breath counts and the algorithm-derived breaths was assessed using the methods described by Bland and Altman’s Sect. 5.3 on replicated data pairs [40]. Agreement was reported as a mean bias with 95% confidence intervals (CIs) and 95% limits of agreement (LOA) and root-mean-square deviation (RMSD) [40].

### Sample size

Sample size estimates for method comparison studies typically depend on the CI required around the LOA, and sample sizes of 100 to 200 provide tight CIs [40]. We estimated that 20 neonates with ten replications each would give the 95% CI of LOA between the first and second methods to be ± 0.76 times the SD of their differences. The study team aimed for a sample size of at least 30 neonates to ensure a diverse population and sufficient replications for tight CIs.

## Results

Between June and August 2019, 35 neonates were enrolled, and 294 clinical observations were completed. We included 130 (44.2%) CO_2_ waveform plots in this analysis (Fig. 2) across 27 neonates, 23 at term (range of gestational age 32–42 weeks). Four preterm neonates, born before 37 weeks gestation, were included. Three of the four preterm neonates received caffeine during their admission. There were on average 4.8 (range 2–9) epochs per subject.

**Fig. 3:**
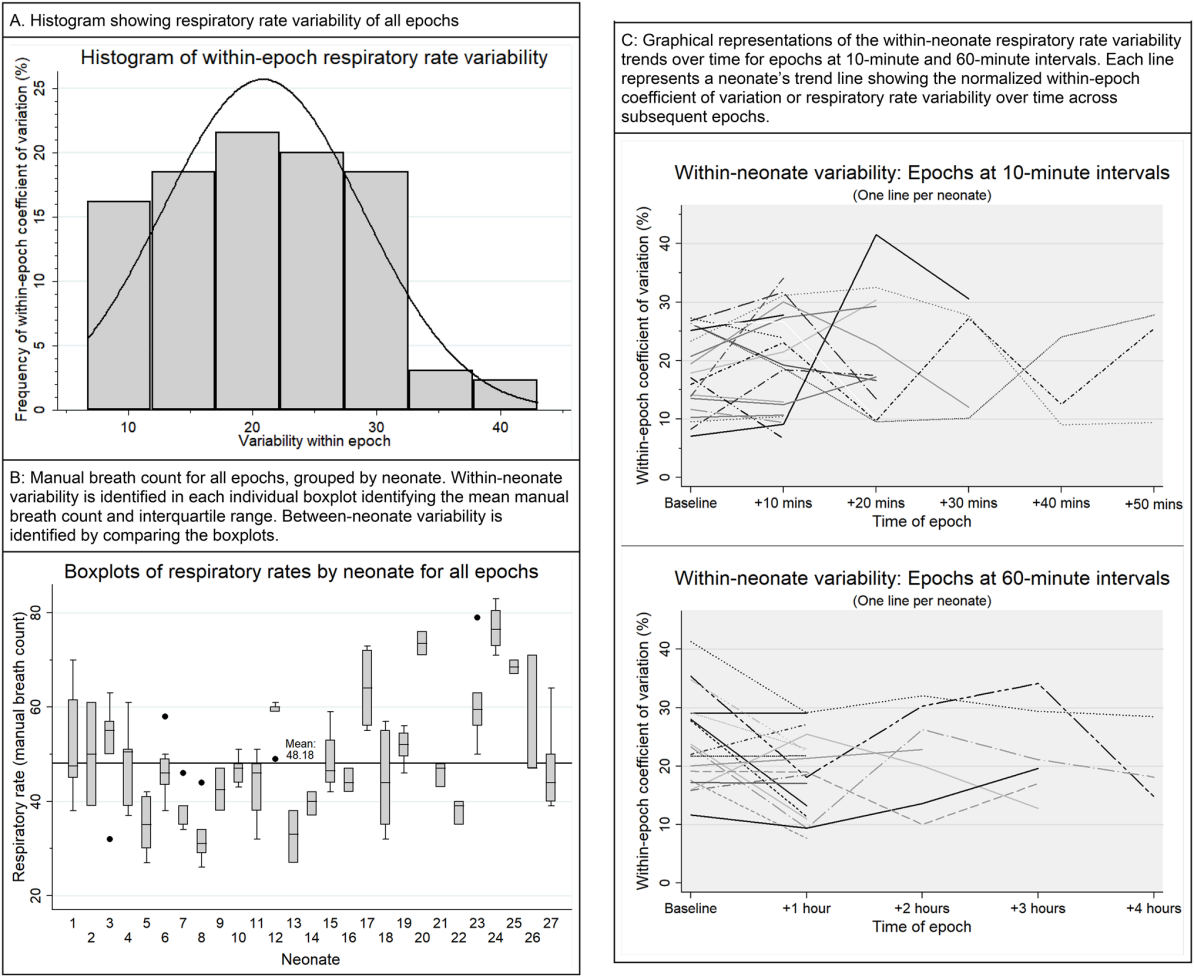
Graphic representations of respiratory rate variability in all epochs (n = 130). **A** Histogram showing respiratory rate variability of all epochs. **B** Manual breath count for all epochs, grouped by neonate. Within-neonate variability is identified in each individual boxplot identifying the mean manual breath count and interquartile range. Between-neonate variability is identified by comparing the boxplots. **C** Graphical representations of the within-neonate respiratory rate variability trends over time for epochs at 10-min and 60-min intervals. Each line represents a neonate’s trend line showing the normalized within-epoch coefficient of variation or respiratory rate variability over time across subsequent epochs

The mean manual breath count was 48 breaths per minute (bpm) (95% CI 31–71) and the median RRV_bm_ was 25.8% (interquartile range (IQR) 22–31.7%; Table 4; Fig. 3A). When grouped by neonate, the mean between-neonate manual breath count was also 48 bpm, while the median RRV_bm_ showed a narrower distribution (12.3%; IQR 9.8–19.4%). The median between-epoch algorithm-derived RRV_bd_ of 25.1% (IQR 21.1–30.8%) was marginally lower than the median manual breath count RRV_bm_.

**Table 4:** Respiratory rate (RR) median and median coefficient of variation between- and within-neonates, as well as between- and within-epochs

		Epochs (n)	Median respiratory or breath rate (interquartile range)	Median respiratory or breath rate coefficient of variation (interquartile range)
Between-neonates	Manual count	130	46.7 (43.1–52.0)	12.3% (9.8–19.4%)
	Algorithm-derived	130	51.2 (45.1–61.8)	17.6% (10.7–24.2%)
Within-neonate	Manual count	130	46.8 (42.5–55.0)	11.5% (6.8–18.9%)
	Algorithm-derived	130	51.0 (45.1–59.3)	17.5% (8.8–24.1%)
Between-epochs	Manual count	130	47.0 (39.0–56.0)	25.8% (22.0–31.7%)
	Algorithm-derived	130	47.5 (40.0–56.0)	25.1% (21.1–30.8%)
Within-epoch	Manual count	N/A	N/A	N/A
	Algorithm-derived	130	50 (41.4–57.1)	20.8% (13.6–27.3%)
*Sub-group*				
Epochs at 10-min intervals	Algorithm-derived (18 neonates)	63	46.1 (38–53)	23.5% (20.5–28.5%)
Epochs at 60-min intervals	Algorithm-derived (19 neonates)	52	50.2 (38.5–60)	28.1% (23.5–36.7%)

Sub-group analysis includes algorithm-derived RR grouped by length of time between epochs. Sub-group analysis excludes neonates with fewer than two time-relevant epochs

Within neonate RRV increased when observations were taken further apart. The median RRV_60bd_ was 4.6% higher compared with the RRV_10bd_ (28.1% (IQR 23.5–36.7%) vs 23.5% (IQR 20.5–28.5%)). A graphical representation of the within-neonate RRV_10bd_ and RRV_60bd_ trends over time showed a marked downward trend in RRV_60bd_ between the baseline and one-hour epochs (Fig. 3C); other time periods did not show this trend.

**Fig. 4:**
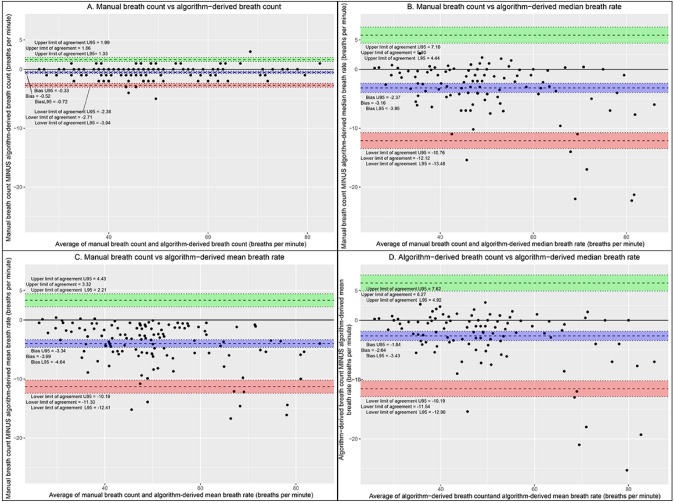
Bland–Altman plots comparing manual breath count vs algorithm-derived breath count (**A**), manual breath count vs algorithm-derived median breath rate (**B**), manual breath count vs algorithm-derived mean breath rate (**C**), and algorithm-derived breath count vs algorithm-derived median breath rate (**D**)

Manual breath count and the algorithm-derived breath count showed minimal bias (− 0.52) and strong agreement (95% lower limit of agreement (LLA) − 2.7, 95% upper limit of agreement (ULA) 1.77, RMSD 1.2; Table 5). However, the manual breath count had a larger bias (at least − 3 bpm), and a larger normalized spread (95% LLA 37.2% and 95% ULA 30.4%) compared with both the algorithm-derived median and mean breath rates and a small bias and tighter spread of 95% LLA and 95% ULA compared with algorithm-derived breath counts (Fig. 4).

**Table 5:** Bland–Altman analysis results comparing manual breath count with algorithm-derived breath counts, median and mean breath rates

	Bias (normalized)	95% Upper/lower limits of agreement	Spread of upper and lower 95% limits of agreement (normalized)	RMSD (normalized)
Manual breath count vs algorithm-derived breath count	− 0.52 (− 1.1%)	− 2.7/1.66	4.37 (9.1%)	1.2 (2.5%)
Manual breath count vs algorithm-derived median breath rate	− 3.16 (− 6.6%)	− 12.12/5.8	17.92 (37.2%)	5.5 (11.4%)
Manual breath count vs algorithm-derived mean breath rate	− 3.99 (− 8.3%)	− 11.3/3.32	14.62 (30.4%)	5.5 (11.4%)
Algorithm-derived breaths vs algorithm-derived median breath rate	− 2.64 (− 5.4%)	− 11.54/6.27	17.82 (36.6%)	5.2 (10.7%)

LOA were calculated based on log-transformed data which were found to be normally distributed as evidenced by the histogram and the Shapiro–Wilk test (S1). We also calculated a non-parametric version of the LOA (S2), which were the values outside which 5% of the observations fell, based on a nonparametric approach for comparing methods [40] which allows the use of dependent data. All these LOA provided qualitatively similar conclusions.

## Discussion

The results, which showed a range of RRV from 11.5 to 25.8% within 60-s epochs, were consistent with previously published research quantifying RRV in neonates, children, and adults, supporting the external validity of the current data while also highlighting challenges when performing device comparison studies [12, 33, 41–43]. This range also suggested the RRV was within a normal range for short-term neonatal monitoring in relatively healthy neonates; no neonates had a deterioration of their condition during or immediately following participation in the study.

Manually counting breaths from a capnogram is a labor-intensive process commonly used in clinical RR measurement. However, due to its limited temporal precision, manual breath counting does not provide for the precise breath durations required when estimating within-epoch mean or median RRV, or breath rate variability. Human observers are not precise enough to identify the exact breath duration differences required to objectively discern accurate variation. An accurate breath identification algorithm can both identify individual breaths and breath lengths from raw waveform data extracted from the capnogram and can be automated. The algorithm used in the current study showed a high level of accuracy for the algorithm-derived breath count as compared to the manual breath count, evidenced by the small bias, tight 95% LLA and ULAs, and a small RMSD across all epochs, and was confirmed by plotting each breath in the capnograms. These results suggested that this algorithm correctly identified individual breaths and could identify breath duration and RRV. Each source of uncertainty (Table 1) increased the challenge in estimating the true RR, and RRV should be considered when comparing MCPM devices.

The marked within-epoch RRV_bd_ that was identified highlights the challenge of performing accurate clinical RR measurements in neonates. Both RR and RRV will be significantly affected by the timing of the start of the epoch analysed. RRV also needs to be reflected in typical clinical decision-making thresholds. A longer measurement period, for example, 60 or 120 s compared to 15 or 30 s, is likely to make the RR more accurate due to the marked short-term, breath-by-breath variability. It is also critical when performing device comparison studies to use the exact same breaths. This requires a high degree of device time synchronization.

Healthy adults have mean RRs that range between 12 and 20 per minute, a 67% difference between lower and upper ‘normal’ values. Healthy neonates have a mean RR of 30 to 60 bpm, a 100% difference [41]. Some healthy neonates have an upper RR range as high as 72 bpm [42]. The substantial neonatal RRV identified in theses results has significant implications for the use of guidelines, setting clinical thresholds, and when comparing RR measurement devices. The United Nations International Children's Emergency Fund (UNICEF) recommended a maximum RRV of no more than two bpm in accuracy for diagnostic device comparisons of acute respiratory infection is not appropriate for neonates [44]. For a neonate with a RR of 70 bpm, this recommendation equates to 2.9% variability, somewhat stricter than any of the within-neonate or between-neonate RRV identified in our results. In an adult, a two bpm difference at ten bpm could be an important difference (20%), but a two bpm difference in a neonate typically breathing at 60 bpm (3.3%) would be less clinically relevant.

Furthermore, there is substantial RRV across individual neonates over time. The algorithm-derived breath counts identified changes in neonatal RRV_10bd_ and RRV_60bd_ and across all epochs. RRV was higher within RRV_60bd_ epochs compared with RRV_10bd_. Previous research looking at pediatric populations has suggested RRV could be used to diagnose sleep apnea–hypopnea syndrome [19]. In adults, within-subject RRV might be useful as a predictor of subsequent intensive care unit admission [18]. In neonates, RRV may be used to improve clinical care, but more research is needed.

A common dilemma encountered when performing studies of RR measurement is the definition of a ‘true’ breath [45]. Various methods have been used to estimate RR across devices that result in the measurement of different respiratory events as breaths. Small ineffectual respiratory efforts are commonly seen in neonates and may not be consistently accepted or rejected as breaths. Capnograms show many different patterns, including small-amplitude ineffective breaths, double breaths (two peaks in a single breath), subsequent breaths starting before the waveform reaches the trough or baseline value, pauses, catch-up rapid breathing, sighs, and sharp rapid cycles as seen with coughs (Fig. 1; Table 3). Further investigation into the identification and inclusion of abnormal respiratory events during respiratory device accuracy comparison studies are needed.

The clinical implication of this high degree of RRV, even in neonates with regular breathing, is that clinicians should be aware of the inherent uncertainty of clinical decisions made based on selected threshold values. It would be advisable to use repeated observations before making critical clinical decisions and ideally to use continuous monitoring devices and values summarized over more than one minute.

When conducting device comparison studies, accuracy thresholds should be adapted to a neonate’s baseline RR. Therefore, we suggest that an accuracy threshold should be normalized as a percentage of the baseline value and not more restrictive than the within-neonate RRV. This aligns with a previous proposal to use a percentage error threshold for LOA to determine the acceptability of a new technique in cardiac measurement and is also relevant when comparing RR measurement technologies [46]. When conducting RR accuracy testing, precise synchronization between investigational and reference devices will ensure that the same breaths are compared between devices. Measuring RR in a calm child, as recommended by WHO, will also minimize variability. However, following the WHO recommendations for RR measurement results in a rounding-down to the nearest breath and assumes the mean breathing rate is the most important clinical variable [47]. Instead, a median RR, unaffected by cough or pause, may be more reflective of the underlying physiological control of breathing and more clinically relevant than a mean breath count over 60 s.

We did not study the full range of RRV in real-world settings, particularly among critically ill neonates. The RRV identified in our study likely under-represents the true RRV present in neonates given the data selection used only capnograms with easy-to-identify breaths in the manual breath count and algorithm-derived breath count processes. Epochs that were excluded from analysis were not evenly distributed across neonates and poor data quality was the most common reason for exclusion. Selecting good quality capnograms likely increased the observed agreement within the Bland–Altman analysis. Expansion of the data quality thresholds for data inclusion would likely result in wider CIs and increased RRV.

The capnogram CO_2_ sampling rate was approximately 20 times per second or 20 Hz which is likely sufficient for an adult breathing at 10 to 20 breaths per minute. However, sampling frequency inaccuracies are more apparent at higher breath rates, such as those seen in distressed neonates (which may exceed 80 breaths per minute). When working with neonates or other populations expected to have high breath rates, sampling rates of 100 Hz, and even as high as > 200 Hz, are suggested [48]. These higher sampling rates would avoid any aliasing effects, enable oversampling to accommodate filtering to remove artifacts, and ensure precision in RRV_bd_ estimation.

In the current study, the resulting algorithm-derived breath count had closer agreement with the manual breath count than either the algorithm-derived mean or median breath rates. This finding highlights the impact that smoothing, averaging, normalizing, or other post-processing procedures may have on RR measurement. Most devices will provide a processed result rather than a count, so consideration as to the impact these post-processing decisions are critically important when evaluating automated devices. The critical question that remains as yet unresolved, is the clinical importance of count or the mean/median as a representation of disease severity? RR is often averaged across multiple breaths, resulting in additional uncertainty when making clinical decisions and when comparing devices.

These results identify the range and sources of RRV found between- and within-neonates, as well as between- and within-epochs. RR is traditionally measured by counting the number of breaths within 60 s. While manual counting may seem to be a practical clinical approach, it has limitations, especially when compared to RR measurement with digital devices. Large within-neonate RRV will also impact the application of RR thresholds in MCPM devices and their clinical applications. For devices that estimate RR, we propose a median value of inter-breath intervals within 60 s to remove any extreme outliers and to minimize the effect of rounding.

## Supplementary Information

Below is the link to the electronic supplementary material.

Supplementary file1 (DOCX 1606 kb)

## Data Availability

Data will be made available on completion of the secondary analyses of the data. We have ethics approval to deposit the data in an open-access repository upon completion of the study.

## Code availability

N/A.
