# Supplementary material for: Assessment of neonatal respiratory rate variability
Source: J Clin Monit Comput. 2022 Mar 25;36(6):1869–79. doi: 10.1007/s10877-022-00840-2 (PMC9637627; doi:10.1007/s10877-022-00840-2)
Supplement: Supplementary file 1 — Supplementary file1 (DOCX 1606 kb) [file 10877_2022_840_MOESM1_ESM.docx]

**Supplementary Material**

| **S1:** Bland-Altman plots comparing log-transformed manual breath count vs log-transformed algorithm-derived breath count (A), log-transformed manual breath count vs log-transformed algorithm-derived median breath rate (B), log-transformed manual breath count vs log-transformed algorithm-derived mean breath rate (C), and log-transformed algorithm-derived breath count vs log-transformed algorithm-derived median breath rate (D). |
| --- |
| **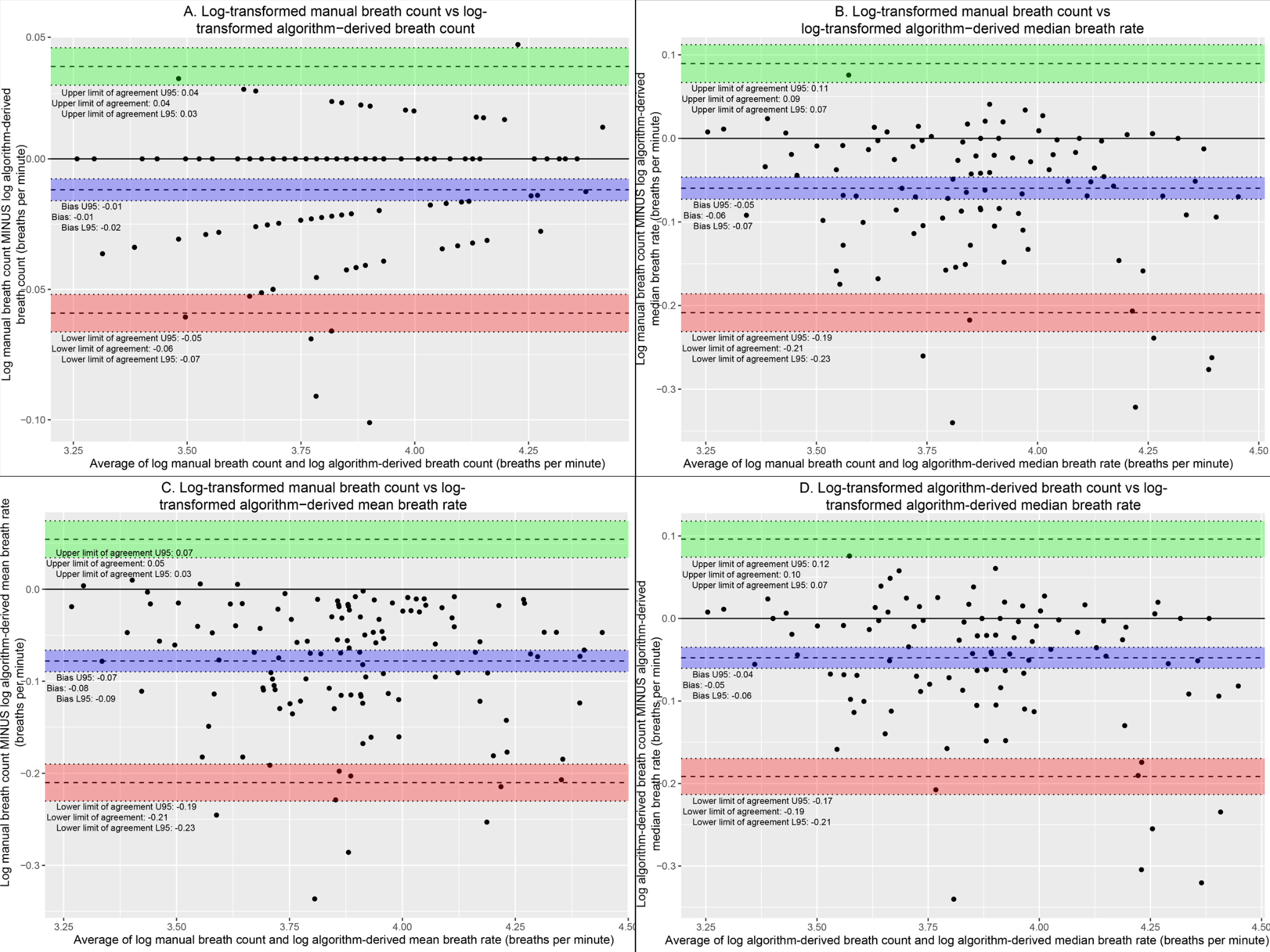** |
| **S2**: Non-parametric version of the Bland-Altman plots with the upper and lower 2.5% of values removed for manual breath count vs algorithm-derived breath count (A), manual breath count vs algorithm-derived median breath rate (B), manual breath count vs algorithm-derived mean breath rate (C), and algorithm-derived breath count vs algorithm-derived median breath rate (D). |
| **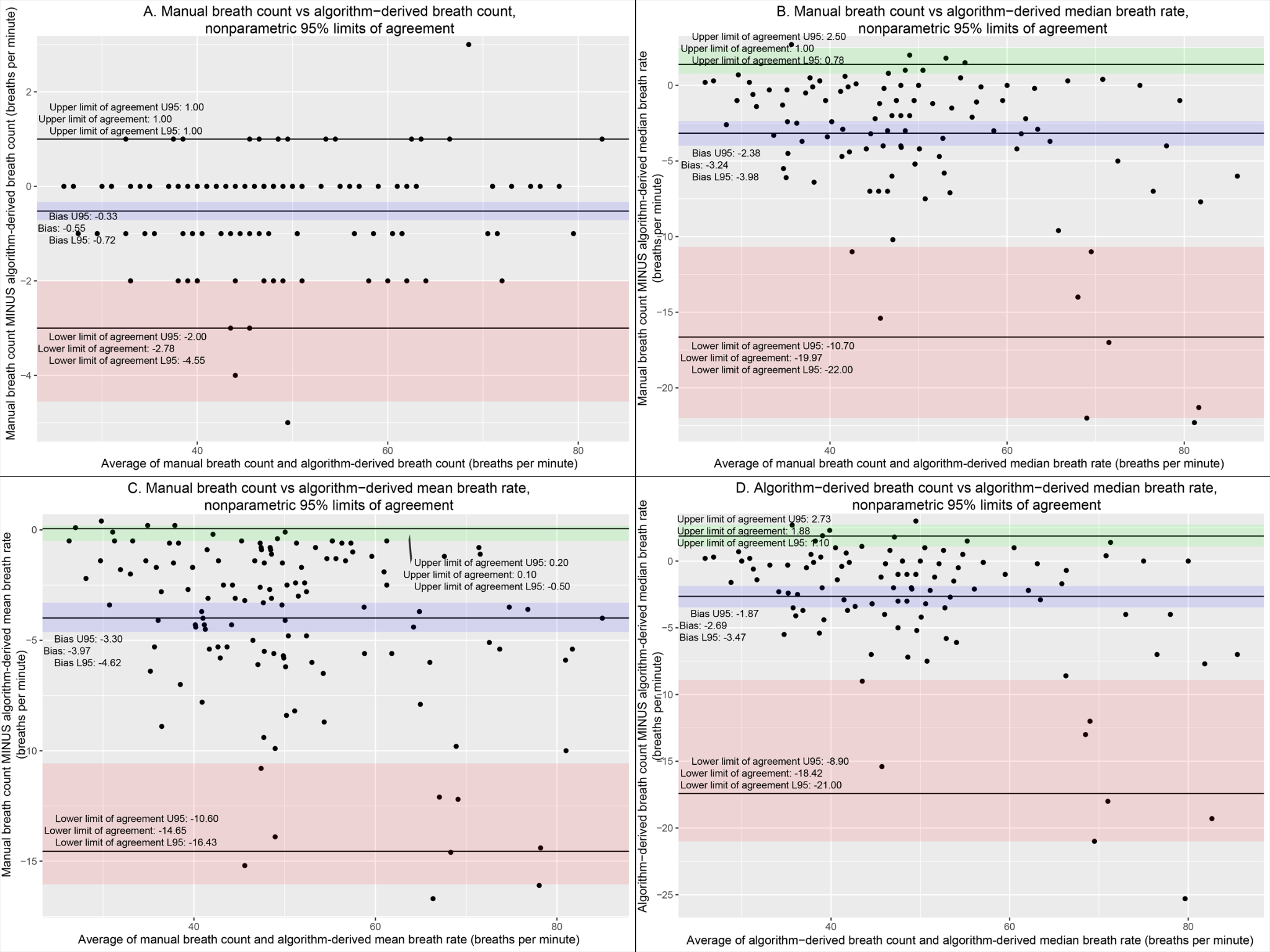** |
